# Biochar Derived from Pineapple Leaf Non-Fibrous Materials and Its Adsorption Capability for Pesticides

**DOI:** 10.1021/acsomega.3c02328

**Published:** 2023-07-11

**Authors:** Assadawoot Srikhaow, Ei Ei Win, Taweechai Amornsakchai, Tanongkiat Kiatsiriroat, Puangrat Kajitvichyanukul, Siwaporn M. Smith

**Affiliations:** † Center of Sustainable Energy and Green Materials and Department of Chemistry, Faculty of Science, Mahidol University, 999 Phuttamonthon Sai 4 Rd, Salaya, Nakhon Pathom 73170, Thailand; ‡ Department of Mechanical Engineering, Faculty of Engineering, Chiang Mai University, 239, Huay Kaew Road, Muang District, Chiang Mai 50200, Thailand; § Department of Environmental Engineering, Faculty of Engineering, Chiang Mai University, 239, Huay Kaew Road, Muang District, Chiang Mai 50200, Thailand; ∥ Sustainable Engineering Research Center for Pollution and Environmental Management, Faculty of Engineering, Chiang Mai University, 239, Huay Kaew Road, Muang District, Chiang Mai 50200, Thailand

## Abstract

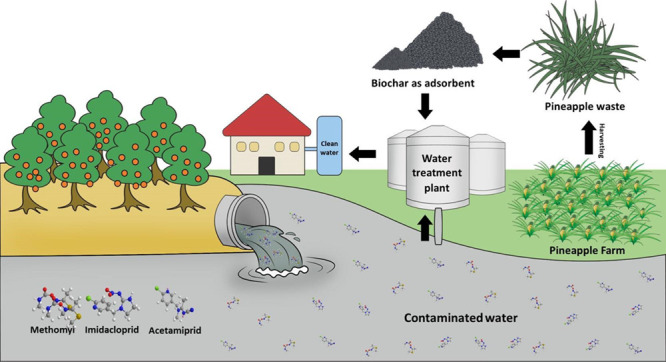

Non-fibrous materials (NFMs) are typically discarded during pineapple leaf fiber processing. The underutilized NFM waste was proposed for use in this work as a raw material for the production of biochar . The removal of pesticides (acetamiprid, imidacloprid, or methomyl) from water was then investigated using the NFM derived biochar (NFMBC). The pseudo-second-order kinetic data suggested chemisorption of pesticide on NFMBC. While acetamiprid or imidacloprid adsorption on NFMBC occurred primarily *via* multi-layered adsorption (best fitted with the Freundlich isotherms), the Sips adsorption isotherms matched with the experimental data, implying heterogeneous adsorption of methomyl on the biochar surface. The adsorption capacities for acetamiprid, methomyl, and imidacloprid are 82.18, 36.16, and 28.98 mg g^–1^, respectively, which are in agreement with the order of the polarity (low to high) of pesticides. Adsorption capacities indicated that the NFMBC preferably removed low-polarity pesticides from water sources. Since pineapple leaves provide fibers and NFMs for materials development, this study should promote an extended agro-waste utilization approach and full-cycle resource management in pineapple fields.

## 1 Introduction

Water contamination arising from the use of cosmetics, pharmaceuticals, synthetic dyes, and pesticides is now receiving considerable public attention due to the detrimental effects of these pollutants on human health and natural ecosystems. These contaminants, having been detected at levels from ng·L^–1^ to several μg·L^–1^ in water courses,^1^ arise from a multitude of sources such as households, hospitals, and industrial and agricultural activities. Agricultural pesticides are of particular concern as they are widely used on a massive scale, and issues related to run-off and accumulation in soil and natural water sources have been previously documented.^2^ Accordingly, regulatory controls and monitoring protocols, in conjunction with effective treatment technologies, are necessary to protect the integrity of ecological systems and the environment.

Acetamiprid and imidacloprid (neonicotinoid pesticides) were recently listed by the U.S. Environmental Protection Agency or USEPA (Decision 2015/95) as being two of seventeen “Watch List Contaminants of Emerging Concern” through monitoring of surface water sources.^3^ They have also been classified by the USEPA as class II (moderately toxic) and class III (slightly toxic). Previous research has indicated that these pesticides may be harmful to human health and other living species such as aquatic animals, birds, and bees.^4^ With their increasingly extensive use in agriculture, acetamiprid and imidacloprid have been detected in environmental water samples from wetlands (acetamiprid, up to 225 μg·L^–1^),^5−7^ agricultural surface water (imidacloprid, 320 μg·L^–1^),^8^ river areas near horticulture and vegetable growing regions (imidacloprid, 4.6 μg·L^–1^), surface water from agricultural regions (imidacloprid, up to 3.29 μg·L^–1^), in agricultural products such as apples (acetamiprid, up to 100 ng·g^–1^, imidacloprid, up to 4.2 ng·g^–1^),^5,9^ cabbage leaf (0.14 mg·kg^–1^),^10^ cantaloupes (acetamiprid, 34.8 ng·g^–1^, imidacloprid, 3.0 ng·g^–1^), cucumbers (acetamiprid, 0.6 ng·g^–1^, imidacloprid, 2.78 ng·g ^–1^),^9^ in soil from seed treatment on winter wheat (imidacloprid, up to 60 μg·L^–1^, after 6 years of repeated application),^11^ and even human urine from areas of China (acetamiprid, up to 0.08 μg·g^–1^ creatinine, imidacloprid, up to 3.84 μg·g^–1^ creatinine) and Japan (acetamiprid, up to 2.01 μg·L^–1^, imidacloprid, up to 2.52 μg·L^–1^). Methomyl (an oxime carbamate insecticide), a class I (restricted use pesticide) according to the USEPA, is harmful to mammals, fish, and aquatic invertebrates.^12^ The high water solubility (57.9 g·L^–1^ at 25 °C) of methomyl and its low sorption affinity to soil are responsible to high possibility to detect methomyl in surface and ground water.^13^ Reported examples of methomyl contamination in soil (0.058 mg·kg^–1^)^14^ and water streams included ground water (up to 10 μg·L^–1^),^15,16^ rivers (3.1 μg·L^–1^), soil (2.18 μg·L^–1^), and strawberry farm canals (30 μg·L^–1^).^17^

Adsorption is one of the effective wastewater abatement methods. In addition to being inexpensive and highly efficient, adsorption processes are scalable and can be integrated into wastewater remediation operation designs.^18^ Activated carbon, graphene-based materials, natural clays, and biochar have all been reported as effective sorbents for removal of organic contaminants from wastewater streams. Biochar materials derived from biomass or agricultural residues have attracted great attention as biofertilizers and sorbents among academic and industrial sectors because biochars can be produced from low-cost, highly abundant biomass or wastes. Heating biomass in a closed system under an oxygen-deficient condition (pyrolysis) leads to the formation of a high-carbon-density material (biochar), which is typically produced from pyrolysis of biomass.^19^ The chemical composition and properties of biochars, including their adsorption activities, depend on the nature of the biomass feedstock. Examples of biochars that have been utilized for removal of pesticides from water are those derived from coconut shell (1 ppm diazinon, 99% removal),^20^ neem tree bark (50 ppm bentazone, ∼51% removal),^21^ peanut shell (20 ppm imidacloprid, 62% removal),^22^ corn straw with P-doping (2 ppm triazine, >96% removal),^23^ and spent grain from brewing (10 ppm pymetrozine, 56% removal).^24^

With an estimated yearly global production of 28.3 million metric tons, pineapple is one of the most extensively consumed tropical fruits.^25,26^ After harvesting and processing, the large quantity of pineapple peels, crowns, cores, and leaves are typically disposed of in landfills or burned in open areas. The latter results in emissions of carbon in the form of gaseous methane (CH_4_) with combustion byproducts (CO_2_ and CO), including the generation of atmospheric pollutants (NO_x_).^26^ Thus, finding alternative uses for pineapple wastes is an attractive endeavor, and previous studies have focused on using these in food science, pharmaceutical development, and materials science (fiber-reinforced^27,28^ and support materials,^28^ and supercapacitors^29^) and sorbents for removal of organic dye pollutants and metal ions from water.^30,31^ To the best of our knowledge, biochars derived from pineapple peel^32,33^ and pineapple leaf^34^ wastes were applied to remove pesticide residues from water. Note that pineapple leaves are abundant post-harvest waste, and the leaves are a source of natural fibers having particular mechanical properties.^28^ Fiber processing extracts fibers from the leaves, separating them from non-fibrous materials (NFMs).^35^ NFMs, which include non-crystalline and small fibers, dust, and other residues, are often tossed away because they are not suitable for either textile or high-quality paper production. Conventionally, NFMs and all crop residues can be used in soil mixes, feed, and biofuel production. Nevertheless, with proper waste management techniques, NFMs should be used to create sustainable, eco-friendly, and value-added products. This study introduces one method for biochar production from pineapple leaf NFMs. After being comprehensively characterized, the biochar (BC) was tested for the adsorption of three widely used pesticides, *i.e.*, acetamiprid, imidacloprid, and methomyl. The adsorption results led to the relationship between important BC properties and the adsorption capacity of pesticides on the BC surface, discussing what makes an effective sorbent for the removal of specific pesticides from water sources. Furthermore, the use of pineapple leaf NFM waste for biochar productions not only reduces waste but also provides a sustainable source of BC.

## 2 Materials and Methods

### 2.1 Chemicals and Materials

In this study, non-fibrous materials (NFMs) are the byproduct after fibers were extracted from dried pineapple leaves.^35^ Non-fibrous material-derived biochar (NFMBC) was produced by apyrolysis of pineapple leaf NFMs at 550 °C for 2 h under a nitrogen atmosphere. The NFMBC used in this work was prepared at the Faculty of Engineering, Chiang Mai University, Thailand. All NFMBC samples were ground and then sieved through a stainless-steel mesh (particle size <177 μm) before utilization. Imidacloprid (Saima Chemical, 70% WG), acetamiprid (Phoenix, 20% w·v^–1^), and methomyl (Hebei Enge Biotech, 97% TC, 970 g·kg^–1^) were of commercial grade and were used without further purification. The properties of pesticides used in this study are given in Table 1. Doubled-distilled water was utilized to prepare all aqueous pesticide solutions.

**Table 1:**
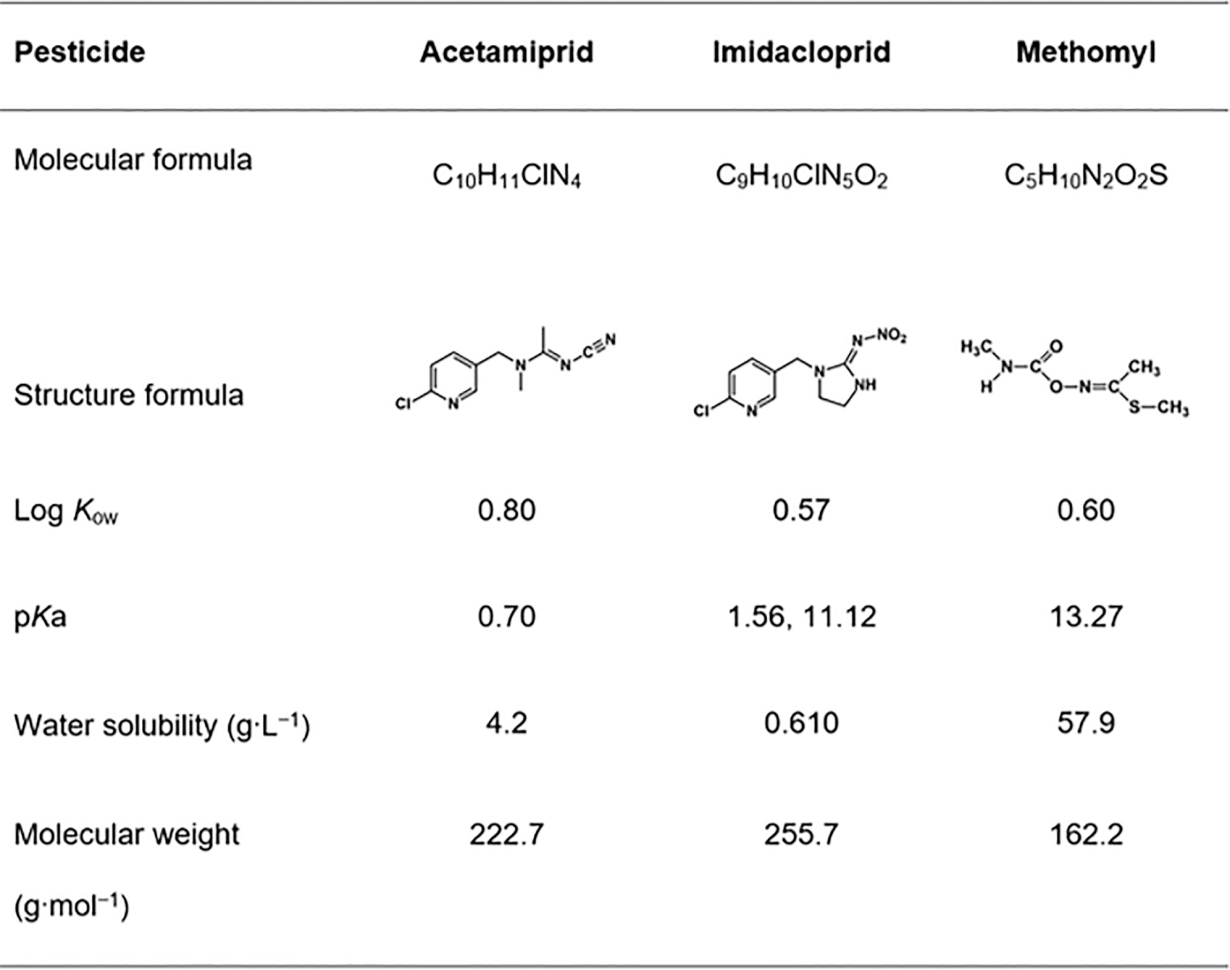
General Properties of Pesticides Studied

### 2.2 Characterization of Biochar

Elemental compositions of biochar samples were analyzed on a CHN analyzer (LECO Corporation, CHNS 628, St. Joseph, MI, USA). Samples were degassed at 200 °C for 12 h prior to N_2_ sorption measurements (ASAP 2026, Micrometrics, Norcross, GA, USA), and the specific surface area was quantified by using the Brunauer–Emmett–Teller (BET) method. Powder X-ray diffraction patterns were recorded on a Bruker D2 phaser diffractometer (Bruker, Billerica, MA, USA) equipped with a Cu Kα radiation source, with diffraction angles (2-Theta) from 10° to 80°. The morphology and surface composition of biochar samples were studied using scanning electron microscopy (SEM, Hitachi, SU800, Tokyo, Japan) and energy-dispersive spectroscopy (EDS), respectively. Fourier transform infrared (FTIR) spectra were recorded using an FTIR spectrometer (Thermo Electron Corporation, Nicolet 6700, Madison, WI, USA) with samples prepared as KBr pellets. Surface elemental composition and functional groups were analyzed by X-ray photoelectron spectroscopy (XPS, AXIS, Ultra DL, Kratos Analytical, Manchester, UK), using a monochromatic Al Kα X-ray excitation source under vacuum conditions. Raman spectra of NFMBC samples were recorded on a Horiba XploRa Plus instrument (Horiba, Kyoto, Japan) with excitation using a 532 nm laser.

### 2.3 Batch Adsorption Experiments for Pesticide Removal

The following protocol was employed for batch adsorption experiments. Polypropylene bottles (500 mL) were filled with 200 mL of 10 ppm aqueous pesticide solution (either acetamiprid, imidacloprid, or methomyl), having a pH between 6.8 and 7.2. Subsequently, biochar (1–7 g·L^–1^) was added to each bottle, and the suspension was agitated using a thermostatic shaker at 150 rpm for 6 h, at varying biochar dosages. Each adsorption experiment was carried out in triplicate. After 6 h, each suspension was filtered through a 0.45 μm cellulose acetate syringe filter, and the absorption spectra of solutions were measured on a UV–vis spectrophotometer (Thermo Scientific, GENESYS 10S, Waltham, MA, USA). The maximum wavelengths (λ_max_) of aqueous imidacloprid, acetamiprid, and methomyl were selected as 270, 246, and 234 nm, respectively. Pesticide concentrations in samples were determined from a calibration plot of concentration versus absorbance (using known pesticide concentrations), based on the Beer–Lambert law.^20^

The removal efficiency of single pesticides and the amount of the pesticide adsorbed at equilibrium (*q*_e_) per unit mass of sorbent were calculated from the following equations:
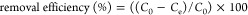
1
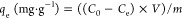
2where *C*_0_ is the initial concentration (ppm) of the pesticide, *C*_e_ is the equilibrium concentration of the pesticide after adsorption (ppm), *m* is the weight of biochar used (g), and *V* is the volume of aqueous pesticide solution (L).

### 2.4 Sorption Isotherms

The isothermal adsorption behavior of each pesticide on biochar sorbents was investigated at initial pesticide concentrations of 10–200 ppm, with a sorbent loading of 5 g·L^–1^ (pH 7, 25 °C). Pesticide adsorption on the biochar was conducted for 8 h to ensure equilibrium was reached. Common adsorption isotherm models [Langmuir, Freundlich, and Sips models (eqs 3–5)] were employed to evaluate the fitness quality of each equation with the sorption data.^36,37^
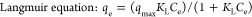
3
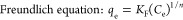
4
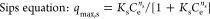
5In the equations above, *q*_max_ is the Langmuir maximum adsorption capacity (mg·g^–1^), whereas *K*_L_ (L·mg^–1^), *K*_F_ (mg^(1–1/*n*)^·g^–1^ L^1/*n*^), and *K*_s_ (L·mg^–1^) are coefficients related to Langmuir adsorption, Freundlich affinity, and Sips model, respectively. In addition, n is the Freundlich empirical constant, while *q*_max,s_ is the Sips maximum adsorption capacity (mg·g^–1^), and *n*_s_ is the Sips isotherm model exponent.

### 2.5 Adsorption Kinetics

Adsorption kinetics at 25 °C were measured at predetermined time intervals over 8 h using a pesticide concentration of 10 ppm and a biochar dosage of 5 g·L^–1^. Three common kinetic models listed in eqs 6–8 were explored, and the one with the best fit with the experimental data, for each pesticide, will be used to describe the adsorption mechanism and sorption rates.^37^
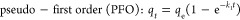
6
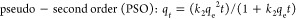
7
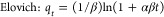
8Notably, *q_t_* is the amount of pesticide adsorbed at a time “*t*” (mg·g^–1^), while *k*_1_ and *k*_2_ are the rate constants for the PFO (min^–1^) and PSO (g·mg^–1^·min^–1^) models, respectively. While α is the initial pesticide adsorption rate for the Elovich model (mg·g^–1^·min^–1^), β is the desorption constant (g·mg^–1^). The rate-controlling phase of the adsorption process was identified via intra-particle diffusion and liquid-film diffusion models determination. Figure S1 depicts additional information and the results of the curve-fitting procedure.

### 2.6 Statistics

To compare the performances of the models used in the adsorption isotherm and adsorption kinetics, the statistical parameter known as the Chi-squared error (χ^2^) was also used to determine the best-fitted model.^38,39^

## 3 Results and Discussion

### 3.1 Sample Characterization

**Figure 1:**
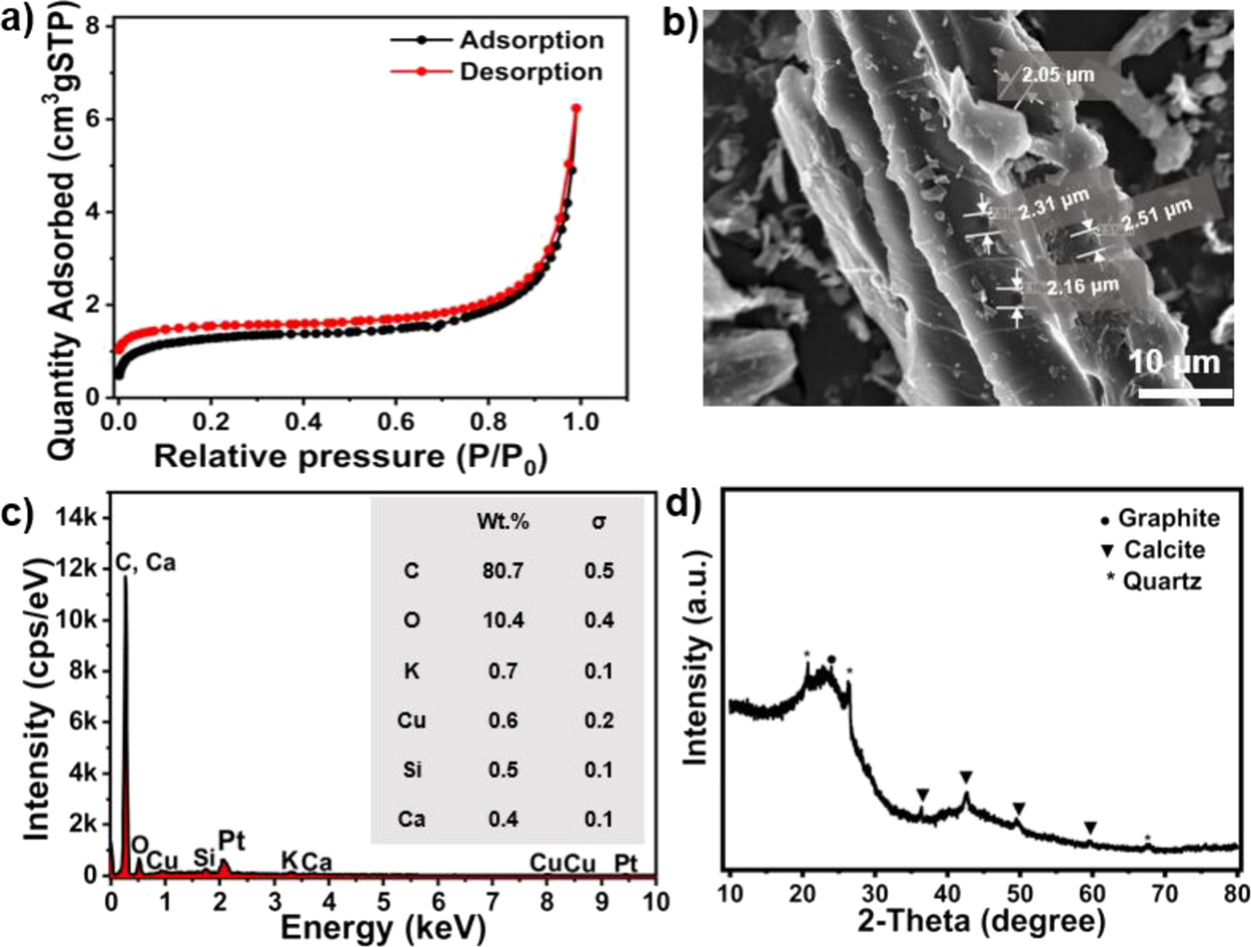
(a) N_2_ adsorption–desorption isotherms of biochar prepared from the non-fibrous material discarded from pineapple leaf fiber (NFMBC); (b) SEM image of NFMBC; (c) EDS spectrum of the NFMBC; and (d) powder XRD pattern of NFMBC. Notably, Pt content in the EDS spectrum originates from the SEM coating prior to measurement.

**Figure 2:**
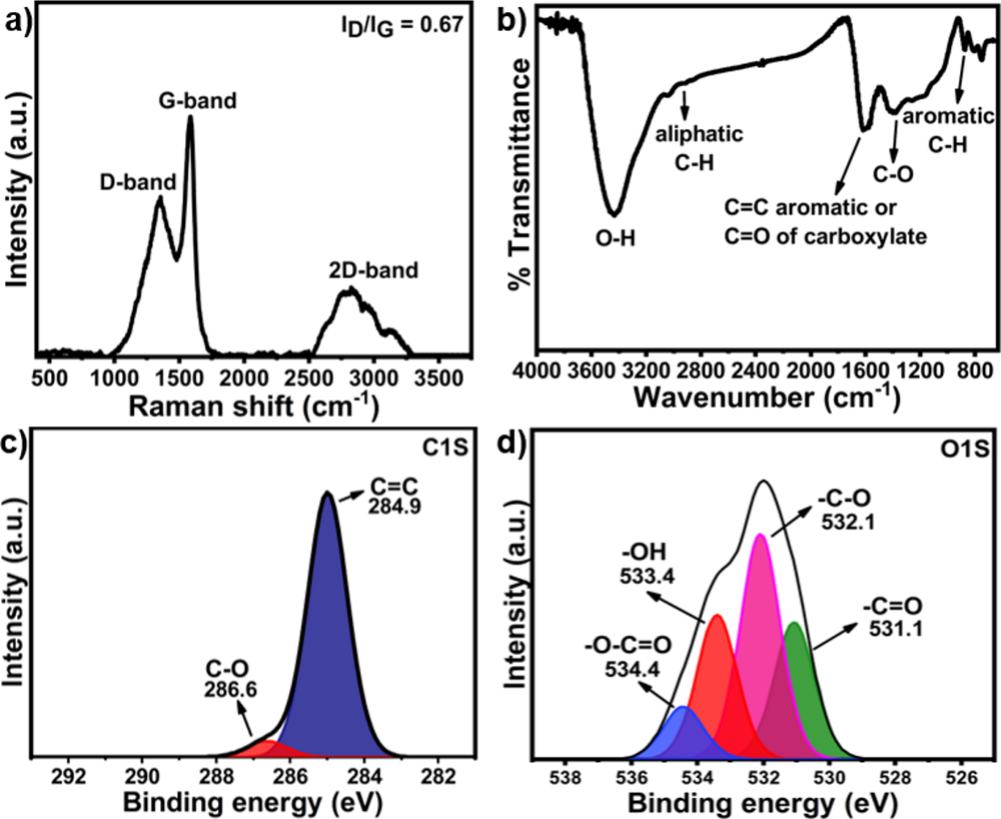
(a) Raman spectrum, (b) FTIR spectrum, (c) XPS C 1s spectrum, and (d) XPS O 1s spectrum of NFMBC. Note that the D and G bands in the Raman spectrum relate to graphitic and defect-containing graphitic structures, respectively.

The NFMBC is composed of C (79.5%), H (2.83%), and N (0.97%), and the carbon content of NFMBC biochar is comparable to that (69–73%) of biochar obtained from pyrolyzed pineapple waste at 500–650 °C.^32,33^Figure 1a displays the nitrogen adsorption–desorption isotherm for NFMBC. Based on the International Union of Pure and Applied Chemistry (IUPAC) classification, the isotherm is type IV and features an H1 hysteresis loop, which is indicative of a mesoporous structure.^40^ The BET surface area (4.65 m^2^ g^–1^) is within the range (2.1–7.3 m^2^ g^–1^) of values obtained for biochar derived from pineapple wastes.^32−34,41^ The average pore size and pore volume of NFMBC biochar were 8.30 nm and 0.0097 cm^3^·g^–1^, respectively. SEM imaging (Figure 1b) revealed that NFMBC has a tube-like structure containing pores with sizes between 2.1 and 2.5 μm. The EDS spectrum in Figure 1c demonstrates that NFMBC primarily comprises carbon (80.7%), which is consistent with the results from combustion analysis, together with oxygen (10.4%) and trace elements K (0.7%), Cu (0.6%), Si (0.5%), and Ca (0.4%). A previous study by Fu et al. reported the presence of trace K and Si components in biochar derived from pineapple peel wastes.^32^ The diffraction profile of NFMBC (Figure 1d) shows a low-intensity broad peak around 23°, indicating an amorphous phase of graphitic carbon. Diffraction peaks corresponding to calcite (CaCO_3_) and quartz (SiO_2_) are also observable and are likely to arise from the parent materials, as previously reported in biochar derived from pineapple peel, corncob,^42^ and red oak feedstocks.^43^ Three distinct D, G, and 2D bands were observed in the Raman spectrum (Figure 2a) in the regions of 1300–1400, 1500–1600, and at 2600 cm^–1^, respectively. While the D band reflects the structural disorder, the G band corresponds to the crystallinity of the sp^2^ carbon material.^44^ The *I*_D_/*I*_G_ intensity ratio value of 0.67 suggested a low defect structure of NFMBC with a high degree of graphitization.^45^ The 2D band, the second-order overtone of the D band, is typically used to determine the graphene layer thickness. As suggested by a previous report, the sharp and intense 2D band reflects the single-layer graphene as the major graphene component^46^ in the carbon-based material studied. Results indicate that the carbon in NFMBC is in the form of multi-layer graphene or graphitic in nature. The FTIR spectrum of NFMBC (Figure 2b) shows an intense peak at 3421 cm^–1^, a characteristic absorption corresponding to O-H stretching vibrations in alcohols, phenols, and carboxylic groups.^47^ The weak band at 2933 cm^–1^ correlates with aliphatic C–H stretching vibrations from cellulose and hemicellulose components, while the intense peak at 1630 cm^–1^ may suggest the presence of the carboxyl group (C=O) and aromatic carbon (sp^2^ graphitic carbon, C=C stretching vibrational mode).^48^ Additionally, the peaks at 1401 and 869 cm^–1^ were ascribed to carboxylate O–C–O asymmetric stretching vibrations and aromatic C–H bending, respectively.^49^ Therefore, the NFMBC material possesses numerous chemically functional groups that are susceptible to non-covalent interactions with pesticide residues, resulting in adsorption.^50^ High-resolution XPS spectra (C 1s and O 1s) for NFMBC are displayed in Figure 2c,d. These reveal that C 1s has two peak components at 284.9 eV (C=C, sp^2^C) and 286.6 eV (C–O)^45^ and that the O 1s peaks confirm the presence of O–C=O, O–H, C–O, and C=O bond types (534.4, 533.4, 532.1, and 531.1 eV, respectively).^51^

### 3.2 Pesticide Adsorption Screening

#### 3.2.1 Sorbent Dosage

**Figure 3:**
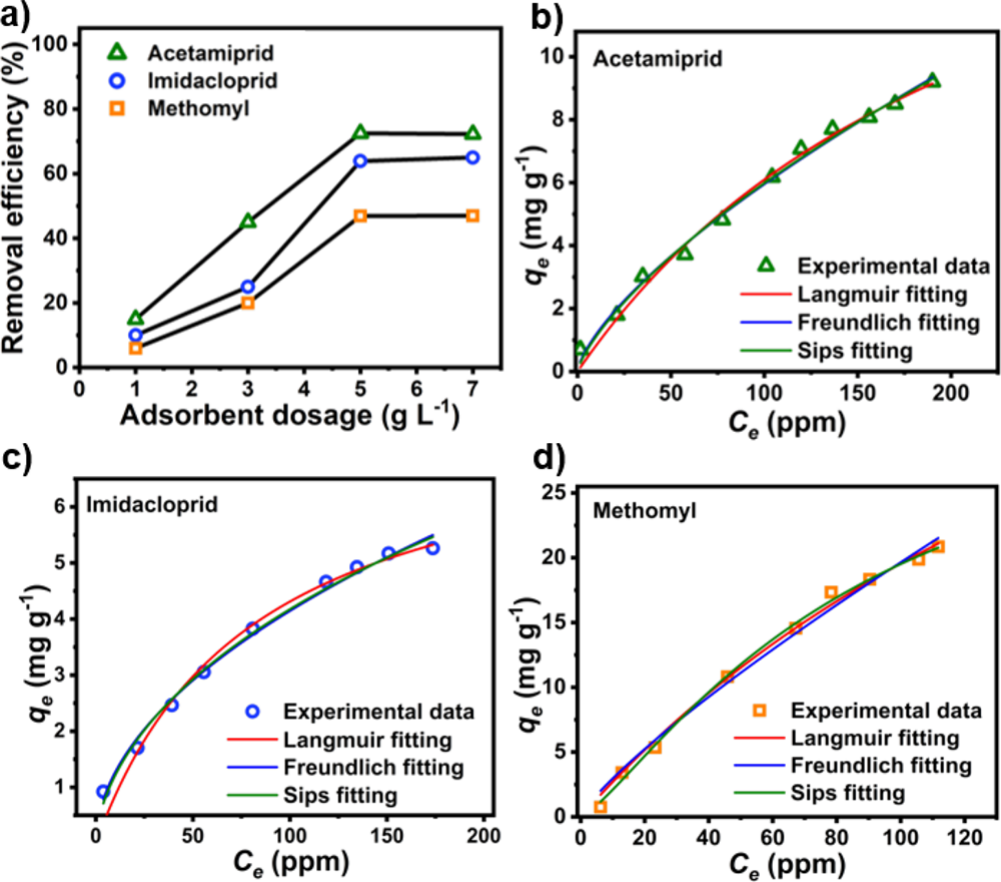
(a) Removal efficiencies of pesticides in aqueous media at varying biochar dosages and adsorption isotherms of (b) acetamiprid, (c) imidacloprid, and (d**)** methomyl on the NFMBC surface. Adsorption conditions used are 200 mL of aqueous pesticide solution, pH = 7, 25 °C, 8 h. Notably, the fitness quality of Langmuir, Freundlich, and Sips isotherm models with the experimental data was examined for each pesticide.

Different dosages (1 to 7 g·L^–1^) of NFMBC sorbent were added into aqueous single pesticide solutions (10 ppm) to study the effect of biochar dosage on pesticide removal. As indicated in Figure 3a, the removal efficiencies of acetamiprid, imidacloprid, and methomyl correlate with sorbent dosage, with greater adsorption at higher dosages presumably resulting from the presence of larger numbers of adsorption sites.^52^

Notably, removal efficiencies for acetamiprid, imidacloprid, and methomyl were the highest (84.39, 64.39, and 46.90%, respectively) at a sorbent dosage of 5 g·L^–1^, and accordingly, this was selected as the suitable dosage for subsequent experiments. Only minimal enhancements at a higher dosage level (7 g·L^–1^) were observed, indicating saturation of the adsorbent sites.^53^

#### 3.2.2 Adsorption Isotherms

Interactions between the adsorbate and the adsorbent are typically described using adsorption isotherms, which indicate the distribution of molecules in a liquid phase and on a solid surface at equilibrium.^45^ Adsorption isotherms of NFMBC for individual pesticides are shown in Figure 3b–d, with the isotherm parameters from fitting the experimental data being provided in Table 2. High correlation coefficient values (*R*^2^, >0.99, Figure 3b–d and Table 2) for all three selected adsorption models made it difficult to decide which model is the best one to describe the sorption behavior of the pesticides on NFMBC biochar. Thus, the χ^2^ values were compared, and the model with the lowest χ^2^ value suggested the best-fitted model for each pesticide. As suggested by the results in Table 2, acetamiprid or imidacloprid adsorption on NFMBC biochar occurred primarily via multi-layered adsorption (Freundlich isotherms have the best fit with experimental data).^54^ On the other hand, methomyl was preferably adsorbed on NFMBC biochar via an energetically heterogeneous, non-uniform surface mode (the Sips model has the best fit with experimental data).^55−59^ Previous studies have also demonstrated the use of the Sips isotherm model to describe the adsorption of dyes and pesticides on biochar and its effectiveness in predicting adsorption behavior at a range of adsorbate concentrations.^55−57^

**Table 2:** Isotherm Parameters for Acetamiprid, Imidacloprid, and Methomyl on NFMBC

model	isotherm parameters	acetamiprid	imidacloprid	methomyl
Langmuir	*q*_max_ (mg·g^–1^)	20.60	7.82	64.76
	*K*_L_ (L·mg^–1^)	0.0042	0.0123	0.0043
	*R*^2^	0.9914	0.9822	0.9948
	χ^2^	0.0801	0.0522	0.3454
Freundlich	*K*_F_ (mg^(1–1/*n*)^·g^–1^·L^1/*n*^)	0.2360	0.3910	0.4460
	*n*	1.4267	0.5126	1.2167
	*R*^2^	0.9931	0.9921	0.9893
	χ^2^	0.0641	0.0231	0.404
Sips	*q*_m,s_ (mg·g^–1^)	82.18	28.98	36.16
	*K*_s_ (L·mg^–1^)	0.0024	0.0114	0.0032
	*n*_s_	0.7551	0.5841	1.2809
	*R*^2^	0.9932	0.9926	0.9965
	χ^2^	0.0711	0.0255	0.2000

#### 3.2.3 Adsorption Kinetics

**Figure 4:**
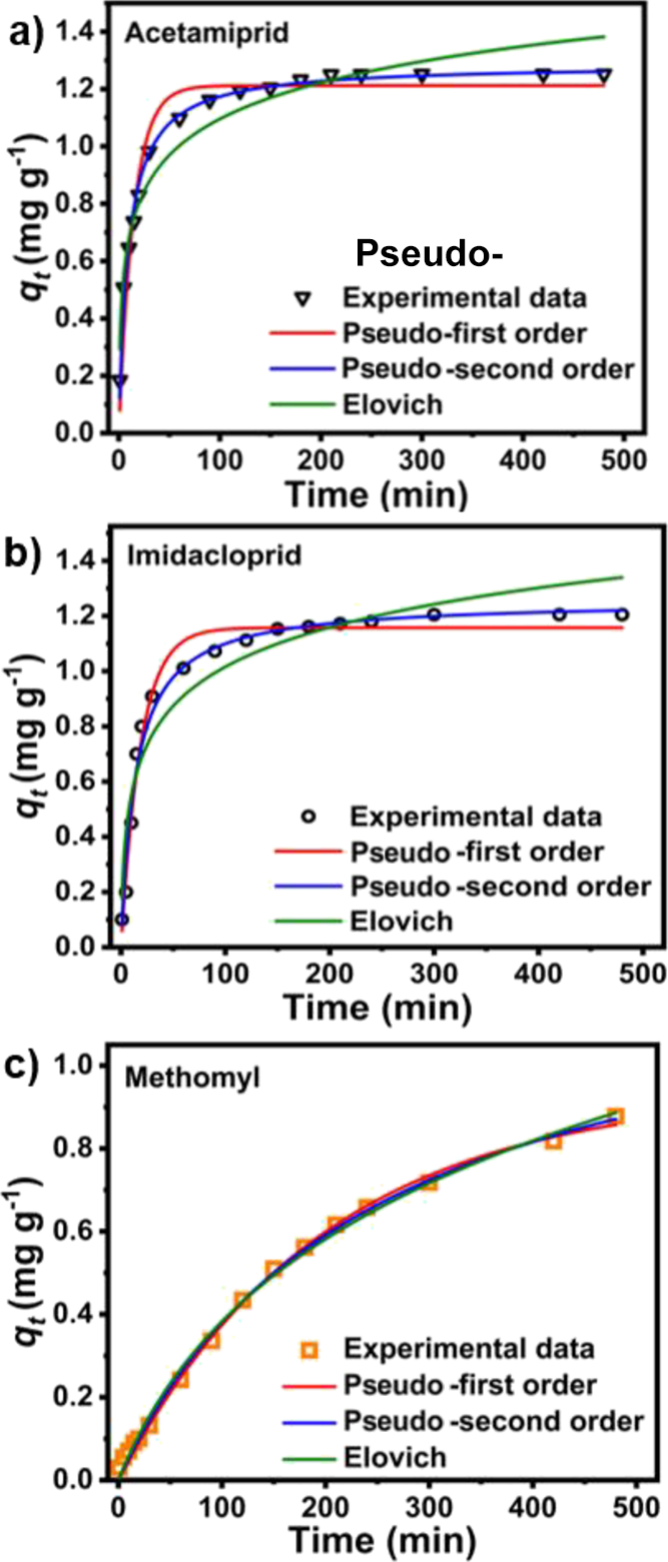
Adsorption kinetics of (a) acetamiprid, (b) imidacloprid, and (c) methomyl on NFMBC, obtained by fitting the experimental data with the pseudo-first-order, pseudo-second-order, and Elovich models.

Kinetic studies of single pesticide adsorption on NFMBC demonstrate that adsorption rates vary by pesticide (Figure 4a–c). Over the first 2 h, adsorption of acetamiprid and imidacloprid on NFMBC occurred faster than that of methomyl. The fast adsorption at the beginning may be caused by rapid mass transfer of a solute from an aqueous pesticide solution to the biochar’s unoccupied sorption sites. Over time, saturation of active sites^60^ reduces the absorption rate, leading to a stage of adsorption equilibrium. To gain a better insight into the adsorption mechanism, non-linear pseudo-first-order (PFO), pseudo-second-order (PSO), and Elovich models were also used to fit the pesticide adsorption data. Curve fitting for the kinetic data and the chrematistic parameters for each model are displayed in Figure 4a–c and Table 3, respectively. It is worth noting that the calculated adsorption capacities (*q*_e,cal_) from the PSO kinetic models are in good agreement with the experimental values (*q*_e,exp_). Moreover, results of kinetic studies also suggest that adsorption kinetics were best described by the PSO kinetic model, with a high correlation coefficient (*R*^2^ > 0.99) and the lowest χ^2^, for single adsorption of pesticides on the NFMBC via prevalent chemisorption processes for all pesticides studied.^61^ These results are consistent with previous studies on adsorption of pesticides by biochar sorbents, in which the pseudo-second-order model has a better fit to the experimental data than others.^52,57,62^ Additionally, the higher values of α relative to β in the adsorption process from the Elovich model indicate that the initial rate of adsorption is higher than the desorption rate,^63^ implying that NFMBC is an effective sorbent for aqueous pesticide removal.

**Table 3:** Kinetic Parameters for the PFO, PSO, and Elovich Models Used in Examining the Adsorption of Acetamiprid, Imidacloprid, and Methomyl on NFMBC^a^

model	parameters	acetamiprid	imidacloprid	methomyl
	*q*_e,exp_ (mg·g^–1^)	1.25	1.20	0.91
PFO	*q*_e,cal_ (mg·g^–1^)	1.21	1.15	0.88
	*k*_1_ (min^–1^)	0.0675	0.0529	0.0051
	*R*^2^	0.9571	0.9812	0.9972
	χ^2^	0.0050	0.0027	0.0026
PSO	*q*_e,cal_ (mg·g^–1^)	1.23	1.24	0.92
	*k*_2_ (g(mg·min)^−1^)	0.0810	00557	0.0031
	*R*^2^	0.9917	0.9901	0.9976
	χ^2^	0.0010	0.0023	0.0022
Elovich	α (mg(g·min)^−1^)	5.4616	4.8257	2.3413
	β (g·mg^–1^)	0.7232	0.2764	0.0062
	*R*^2^	0.9503	0.9223	0.9963
	χ^2^	0.0058	0.0112	0.0030

a *q*_e,exp_ is the experimental adsorption capacity, while *q*_e,cal_ is the calculated adsorption capacity.

Next, intra-particle diffusion and liquid-film diffusion models were applied to study the rate-controlling mechanism for adsorption of pesticides on NFMBC. The fitting curves are displayed in Figure S1a, b, and the calculated parameters are summarized in Table 4. The correlation coefficient (*R*^2^) greater than 0.99 suggested that liquid-film diffusion is the main rate-limiting step, which involves the pesticide transport from the bulk solution to the biochar external surface.^64^ More importantly, the fitting curves have non-zero *y*-intercept values, implying complicated processes during pesticide adsorption on NFMBC. The large thickness of the film diffusion layers (suggested by the *C* values, Table 4) may relate to the higher adsorption capacities for acetamiprid and imidacloprid over NFMBC, compared to that for methomyl. The negative *C* values (for methomyl adsorption) can be ascribed to the effects of film diffusion and surface reaction control.^65^

**Table 4:** LFD and IPD Model Kinetic Parameters for Acetamiprid (ACE), Imidacloprid (IMI), and Methomyl (MET) Adsorption on NFMBC

pesticides	LFD model		IPD model		
	*K*_lfd_ (min^–1^)	*R*^2^	*k*_ipd_ (mg·g^–1^ min^0.5^)	*C* (mg·g^–1^)	*R*^2^
acetamiprid	0.0201	0.9907	0.0621	0.4995	0.9050
imidacloprid	0.0174	0.9908	0.0713	0.3359	0.7870
methomyl	0.0052	0.9975	0.0477	–0.0955	0.9827

#### 3.2.4 Adsorption Mechanism

**Figure 5:**
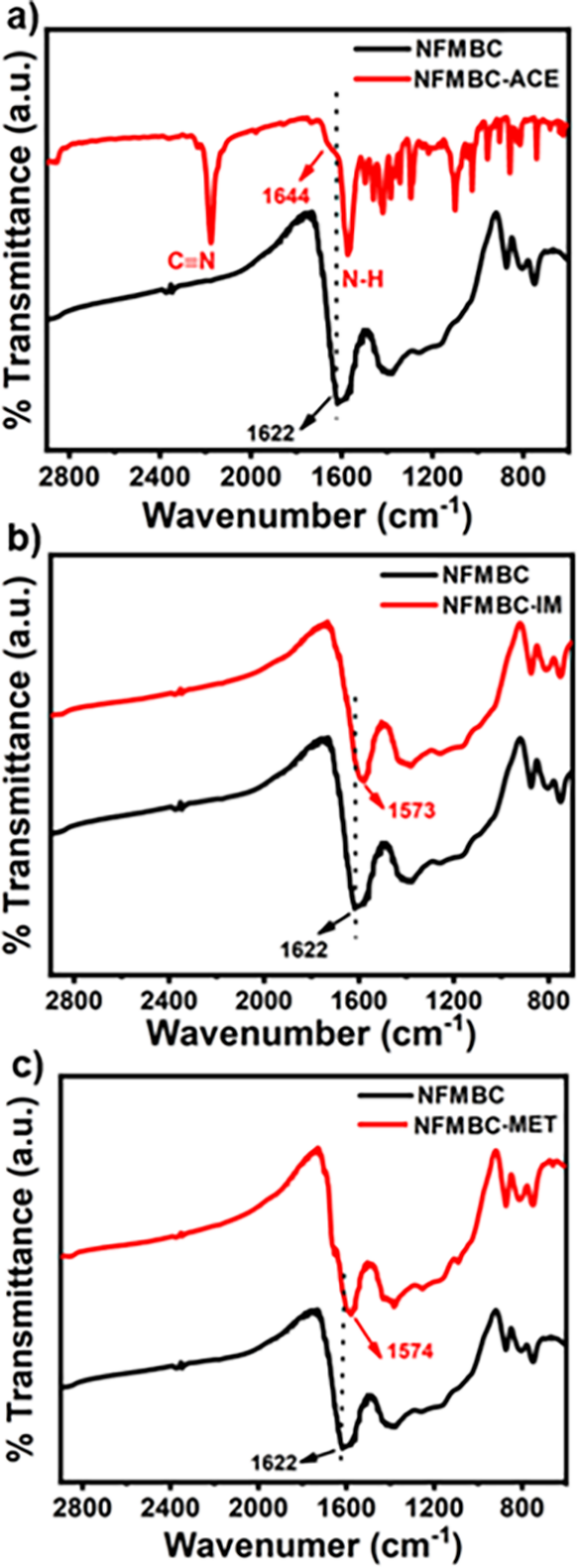
FTIR spectra of NFMBC (black) and the spent NFMBC samples (red) after treatment with (a) acetamiprid (ACE), (b) imidacloprid (IM), and (c) methomyl (MET).

Previous studies have highlighted the importance of non-covalent interactions such as electrostatics, π+−π electron donor–acceptor (EDA) interactions, hydrogen bonding, and hydrophobic effects on the sorption of pesticides by carbon-based materials.^52,66^ As indicated earlier, NFMBC has a sufficient surface area and porosity to provide available active sites for binding with pesticide molecules, and oxygen-containing functional groups (O–H, C=O, −C–C=O, and −C–O) and aromatic carbon (C=C) are available on the surface to facilitate pesticide/surface interactions. As seen in the FTIR spectrum in Figure 5a, vibration peaks from acetamiprid molecules after adsorption with biochar appear at 2200 (CN) and 1568 cm^–1^ (N–H in amines).^67^ Furthermore, FTIR spectra of NFMBC after adsorption (Figure 5a–c) also demonstrate that broad peaks attributed to C=C and C=O (ca. 1622 cm^–1^) shift to a lower wavenumber (Figure 5b,c) on adsorption of imidacloprid and methomyl, whereas those peaks shift to a higher wavenumber at the small shoulder peak (1644 cm^–1^, Figure 5a) overlapping with the peak corresponding to N–H in biochar after adsorption with acetamiprid. This peak shift implies that π+−π EDA interactions between aromatic moieties (C=C) in the biochar with pyridine rings (in acetamiprid and imidacloprid) and the amide N–H (in methomyl) may be occurring. Hydrogen bonding interactions between carboxylic groups (C=O) on the biochar surface and pesticide molecules are also feasible. Normally, pesticides exhibiting high log *K*_ow_ values (low polarity) adsorb more favorably on biochar.^68−70^ All of the studied pesticides are polar (*K*_ow_ < 1); nevertheless, it is expected that the order of adsorption capacities for pesticides on NFMBC should follow the trend acetamiprid (log *K*_ow_ = 0.80) > methomyl (log *K*_ow_ = 0.60) > imidacloprid (log *K*_ow_ = 0.57). Based on the Sips maximum adsorption capacity (*q*_max,s_), the order of *q*_max,s_ values was determined for acetamiprid (82.18 mg·g^–1^) > methomyl (36.16 mg·g^–1^) > imidacloprid (28.98 mg·g^–1^), matching log *K*_ow_ values. As a result, pesticide polarity affects adsorption, with low-polarity pesticides adsorbing better on NFMBC surfaces.

#### 3.2.5 Comparison with Other Sorbents

In this work, the Langmuir adsorption capacities of acetamiprid, methomyl, and imidacloprid on the NFMBC (*S*_BET_ = 4.65 m^2^·g^–1^) were 20.60, 64.76, and 7.78 mg·g^–1^, respectively.

As indicated in Table 5, NFMBC adsorbs acetamiprid, methomyl, and imidacloprid more effectively than eucalyptus wood biochar, natural clays, carbon xerogel, peach stone activated carbon, magnetic sludge biochar, polyethylene microplastics, and magnetic copper-based metal–organic frameworks (MOFs).

**Table 5:** Langmuir Adsorption Capacities of NFMBC Compared with Other Adsorbents

adsorbent	pesticide	temp (°C)	adsorption capacity (mg·g^–1^)	surface area (m^2^ g^–1^)	ref.
biochar derived from pineapple leave non-fibrous materials	acetamiprid	25	20.60	4.65	this work
eucalyptus wood biochar	acetamiprid	25	4.78	4.02	(57)
bentonite	acetamiprid	25	9.17	23.76	(71)
kaolin	acetamiprid	25	7.75	22.71	
biochar derived from pineapple leave non-fibrous materials	methomyl	25	64.76	4.65	this work
eucalyptus wood biochar	methomyl	25	32.42	4.02	(57)
carbon xerogel	methomyl	25	15.20	212.20	(72)
peach stone activated carbon	methomyl	25	7.78	112.0	(73)
biochar derived from pineapple leave non-fibrous materials	imidacloprid	25	7.82	4.65	this work
CoFe_2_O_4_-sludge biochar	imidacloprid	25	5.57	150	(74)
polyethylene microplastics	imidacloprid	25	2.63		(75)
magnetic copper-based MOF	imidacloprid		3.22	250.33	(76)

While the surface area of NFMBC is lower than that of clay, MOF- and carbon-based sorbent NFMBC exhibits higher pesticide adsorption capacities, possibly due to the ability of biochar to undergo a high degree of swelling in aqueous environments, suggested by previous work.^77^ Other reports also stated that biochar was used as an additive to boost the water retention capability of various polymer composites.^78−80^

This study demonstrates that NFM from pineapple leaf fiber processing can be used to produce waste-derived biochar, an effective pesticide biosorbent. Notably, the cost-effectiveness of sorbents also depends on their lifecycle in the wastewater remediation unit, i.e., sorbent recyclability or regenerability. According to the literature, sorbent regeneration methods^81^ include thermal regeneration,^82^ solvent regeneration,^83^ chemical precipitation,^84,85^ microwave irradiation regeneration, and supercritical fluid regeneration. Enhanced performance of an exhausted biochar sorbent in subsequent runs and extended lifecycle of the biochar sorbents would minimize the problems associated with the disposal of exhausted biochar and reduce the need to harvest and transport fresh feedstock to produce new biochar batches. Ineffective spent sorbents, which are classified as hazardous waste, are frequently treated in well-controlled incineration systems^86,87^ or specific biological treatments,^88,89^ avoiding the formation of toxic air pollutants, while ash products can be disposed of in landfills. Nevertheless, energy-intensive and complex protocols involving expensive chemicals rendered the utilization of biochar materials as sorbents in wastewater treatment plants less economical. Depending on the application, scale, location (rich or limited agricultural waste resources), transportation costs, labor costs, and biochar production system, all relevant costs should be determined in a full-cycle assessment to evaluate whether the regeneration expenses, in specific cases, offset the cost for disposal of foul biochar and the cost of biochar new batches.

## 4 Conclusions

The biochar derived from pineapple leaf fiber processing byproduct (NFMBC) can effectively remove aqueous pesticides, acetamiprid, imidacloprid, and methomyl. Adsorption and kinetic analyses suggested the multi-layered adsorption of acetamiprid or imidacloprid and non-uniform adsorption of methomyl on NFMBC (as suggested by the χ^2^ values). The best-fitted pseudo-second-order model suggests that pesticide adsorption is controlled primarily by chemisorption and that liquid-film diffusion is the main rate-limiting step. The maximum adsorption capacities on NFMBC of acetamiprid > methomyl > imidacloprid correlate well with the polarity of the pesticides studied (polarity: acetamiprid < methomyl < imidacloprid). The NFMBC, a waste-derived biochar, should be suitable for remediation of water contaminated with pesticides and other organic pollutants, promoting the feasibility of wastewater treatments with such a low-cost sorbent material. Further studies are required to explore suitable regeneration methods to enhance the lifecycle of the biochar and the adsorption capacity of pesticides on the exhausted biochar and to promote the sustainability of biochar for wastewater remediation applications.

## Supporting Information Available

Intra-particle diffusion (IPD) and liquid-film diffusion (LFD) models employed to examine the adsorption rate-controlling mechanism of acetamiprid (ACE), imidacloprid (IMI), and methomyl (MET) on NFMBC (PDF)
