# Supplementary material for: Biochar Derived from Pineapple Leaf Non-Fibrous Materials and Its Adsorption Capability for Pesticides
Source: ACS Omega. 2023 Jul 11;8(29):26147–57. doi: 10.1021/acsomega.3c02328 (PMC10373191; doi:10.1021/acsomega.3c02328)
Supplement: Supplementary file 1 — ao3c02328_si_001.pdf [file ao3c02328_si_001.pdf]

Supplemental Information

Biochar derived from Pineapple Leaf Non-fibrous Materials and its Adsorption Capability for Pesticides

Assadawoot Srikhaow [1,a], Ei Ei Win [1], Taweechai Amornsakchai [1], Tanongkiat Kiatsiriroat [2], Puangrat Kajitvichyanukul [3,4], and Siwaporn M. Smith [1,*]

[1] Center of Sustainable Energy and Green Materials and Department of Chemistry, Faculty of Science, Mahidol University, 999 Phuttamonthon Sai 4 Rd, Salaya, Nakhon Pathom 73170, Thailand.

[2] Department of Mechanical Engineering, Faculty of Engineering, Chiang Mai University 239, Huay Kaew Road, Muang District, Chiang Mai 50200, Thailand.

[3] Department of Environmental Engineering, Faculty of Engineering, Chiang Mai University 239, Huay Kaew Road, Muang District, Chiang Mai 50200, Thailand.

[4] Sustainable Engineering Research Center for Pollution and Environmental Management, Faculty of Engineering, 239, Huay Kaew Road, Muang District, Chiang Mai University, Chiang Mai, 50200, Thailand.

Corresponding Author: Siwaporn Meejoo Smith

Center of Sustainable Energy and Green Materials and Department of Chemistry, Faculty of Science, Mahidol University, 999 Phuttamonthon Sai 4 Rd, Salaya, Nakhon Pathom 73170, Thailand; https://orcid.org/0000-0001-7571-3636;
Email: siwaporn.smi@mahidol.edu

[a] Department of Earth Resources Engineering, Kyushu University, Fukuoka, 819-0395, Japan

Adsorption rate-controlling mechanism

The adsorption-rate-control mechanism of pesticides on NFMBC was examined using two models, the intra-particle diffusion (IPD) model (Eq. S1) and the liquid film-diffusion (LFD) model (Eq.S2)

$$qt = k_{ipd} \cdot t^{1/2} + C \qquad \text{(S1)}$$

$$\ln(1\text{-}F) = -k_{lfd} \cdot t \qquad \text{(S2)}$$

$$\text{where } F = q_t/q_e \qquad \text{(S3)}$$

k_{ipd} is the rate constant of the IPD model (mg g^{-1} $min^{0.5}$), C is the concentration (mg g^{-1}) which corresponds to the boundary layer thickness, and k_{lfd} is the equilibrium fractional attainment (min^{-1}).[1,2]

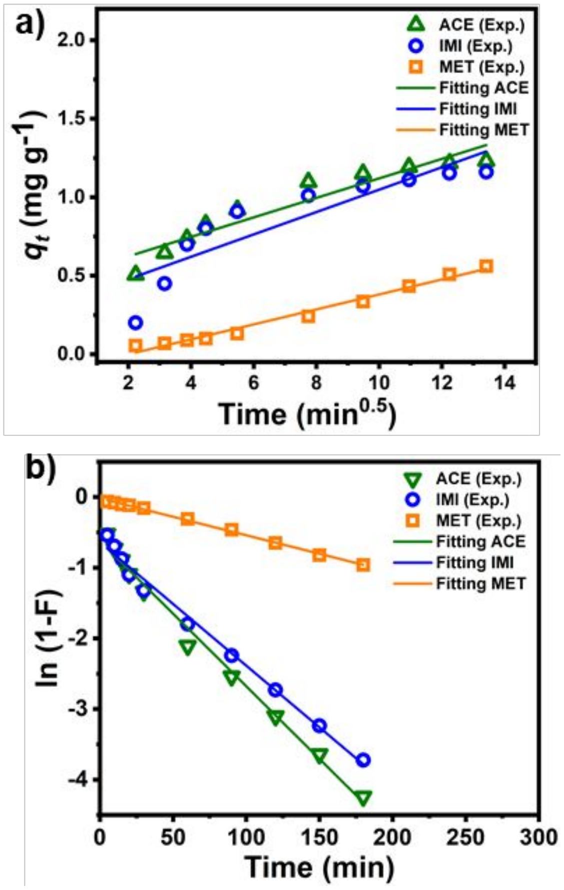

Figure S1: (**a**) IPD model and (**b**) LFD model fitting for the adsorption of acetamiprid (ACE) imidacloprid (IMI) and methomyl (MET) on NFMBC.

References

1. Moussavi, G.; Hosseini, H.; Alahabadi, A. The investigation of diazinon pesticide removal from contaminated water by adsorption onto NH_4Cl-induced activated carbon. *Chem. Eng. J.* **2013**, *214*, 172–179.
2. Wang, F.; Sun, W.; Pan, W.; Xu, N. Adsorption of sulfamethoxazole and 17β-estradiol by carbon nanotubes/$CoFe_2O_4$ composites. *Chem. Eng. J.* **2015**, *274*, 17–29.
